# Associations of ADHD and Borderline Personality Disorder with Suicidality in Adolescents: Additive and Interactive Effects

**DOI:** 10.3390/jcm15010224

**Published:** 2025-12-27

**Authors:** Bartłomiej Sporniak, Przemysław Zakowicz, Monika Szewczuk-Bogusławska

**Affiliations:** 1 Department of Psychiatry, Wroclaw Medical University, 50-367 Wroclaw, Poland; monika.szewczuk-boguslawska@umw.edu.pl; 2 Department of Genetics in Psychiatry, Poznan University of Medical Sciences, 61-701 Poznań, Poland; 3 Department of Neuroengineering and Space Medicine, University of Zielona Góra, 65-516 Zielona Góra, Poland

**Keywords:** adolescence, suicidality, suicide risk assessment, comorbidity, suicidal behaviors

## Abstract

**Background/Objectives**: Suicidal behaviors are a major clinical concern in adolescents, particularly among those with disorders marked by emotion dysregulation and impulsivity. Although attention-deficit/hyperactivity disorder (ADHD) and borderline personality disorder (BPD) each heighten suicide risk, little is known about whether their occurrence confers additive or interactive effects in youth. This study examined whether ADHD and BPD diagnoses show additive or interactive associations with the suicide risk in adolescents. **Methods**: In this cross-sectional observational clinical study, the sample included 108 Polish adolescents (66.7% female; aged 13–17 years) recruited from inpatient and outpatient psychiatric settings (Independent Public Healthcare Facility, Children and Youth Treatment Center in Zabór, the Youth Sociotherapy Center No. 2 in Wrocław, and the District Educational Center in Jerzmanice-Zdrój (Poland)). The data collection for our study was conducted between May 2024 and July 2025. Diagnoses and suicide risk were assessed using the Mini International Neuropsychiatric Interview for Children and Adolescents (MINI-KID 7.02). Associations of ADHD and BPD with suicide risk were tested using linear and logistic regression models while accounting for age, sex, the current depressive episode, and the use of psychiatric medications. **Results**: Unadjusted analyses revealed significant main, but not interactive, associations of BPD and ADHD with suicide risk. When covariates were included in the model, BPD remained strongly associated with suicidality severity and with the presence of any suicide risk (adjusted OR = 7.00, 95% CI [1.55–31.57]), whereas the association between ADHD and suicidality was attenuated and did not reach conventional levels of statistical significance (adjusted OR = 3.48, 95% CI [0.93–13.08]). No statistically detectable ADHD × BPD interaction was observed. Estimates for ADHD were directionally consistent across models but characterized by wide confidence intervals. **Conclusions**: Adolescents with BPD appear to be at particularly high risk of suicide and should receive focused assessment, safety planning, and early intervention as part of routine care. In contrast, suicidality among adolescents with ADHD appears to be influenced by co-occurring clinical conditions, and its independent association with suicide risk remains statistically uncertain after adjustment. Clinicians should therefore remain alert to suicidality in youth with ADHD, while paying particular attention to accompanying symptoms and comorbid diagnoses that may further increase risk.

## 1. Introduction

Self-harm and suicidal behaviors represent major public health concerns, particularly among adolescents struggling with emotion regulation and impulse control [1,2]. Two psychiatric conditions that prominently feature these difficulties are attention-deficit/hyperactivity disorder (ADHD) and borderline personality disorder (BPD). ADHD, affecting approximately 8% of children and adolescents [3], is characterized by persistent patterns of inattention and/or hyperactivity–impulsivity [4]. Children and adolescents with ADHD frequently experience impairments across multiple domains, including peer relationships, social skills, and social cognition [5]. Moreover, ADHD in adolescents often co-occurs with other psychiatric disorders, most commonly behavior and anxiety disorders [6,7]. Extensive meta-analyses have confirmed that ADHD is associated with suicidal ideation and behaviors across developmental stages [8,9].

BPD, in contrast, involves pervasive instability in affect regulation, self-image, and interpersonal relationships [4]. Although typically diagnosed in adulthood, its core features (emotional instability, impulsivity, and interpersonal sensitivity) often emerge during adolescence [10] and are strongly linked to suicidal ideation and attempts [11,12]. ADHD and BPD often co-exist. Epidemiological studies in adults have shown a 27–33% overlap between BPD and ADHD diagnoses [13,14]. Data in developmental populations remain scarce, although clinical studies in developmental samples estimate comorbidity rates ranging from 19% [15] to 33% [16], depending on sample characteristics.

Research further indicates that the combined presence of ADHD and BPD is associated with heightened impulsivity and more severe behavioral disorders [17,18]. This overlap has been linked to increased severity across nearly all comorbid psychiatric symptoms, including aggression, self-harm, suicidal ideation, and substance use [16]. Moreover, recent evidence suggests that the association between ADHD and self-harm or suicidal behaviors may be largely driven by overlapping BPD symptoms [19], emphasizing the importance of considering borderline pathology in suicide risk assessment. Despite these converging lines of evidence, virtually no studies have directly examined whether ADHD and BPD diagnoses exert additive or interactive effects on suicidality specifically in adolescents. Existing research provides only indirect insights: most studies have primarily focused on prevalence or comorbidity patterns [15,16,17], without assessing whether the combined presence of ADHD and BPD confers multiplicative rather than merely additive risk. Therefore, the present study aimed to examine whether ADHD and BPD diagnoses show additive or interactive associations with suicide risk in adolescents.

Clarifying this relationship is clinically important, as it may inform risk assessment and intervention strategies in complex youth psychopathology. We hypothesized that both ADHD and BPD would be associated with higher suicidality scores because both disorders are characterized by mechanisms strongly linked to suicidal behavior, including emotion dysregulation, impulsivity, and interpersonal distress [8,11,20]. Additionally, prior evidence showing increased impulsivity, behavioral dysregulation, and self-harm severity in individuals with co-occurring ADHD and BPD [16,17] suggests that overlapping vulnerabilities may interact to produce elevated suicide risk.

From a clinical perspective, an additive effect would indicate that ADHD and BPD each contribute independently to suicide risk, such that their combined presence results in a cumulative increase proportional to their individual effects. In contrast, an interactive effect would imply a synergistic process, whereby the joint presence of ADHD and BPD amplifies suicide risk beyond the sum of their separate contributions, potentially reflecting mutually reinforcing mechanisms such as impulsivity, affective instability, and interpersonal stress.

Importantly, not all statistically detectable interactions are necessarily clinically meaningful. In the context of multifactorial suicide risk, an interaction would be considered clinically relevant only if it accounted for a non-trivial incremental proportion of explained variance (e.g., ≥2–3% additional R^2^ beyond main effects), or if it identified a qualitatively distinct high-risk subgroup with clear implications for assessment or intervention. This distinction motivated our examination of both additive and interactive associations between ADHD and BPD in relation to adolescent suicidality.

We formulated two hypotheses: (1) ADHD and BPD diagnoses would each be associated with higher levels of suicidality. (2) Adolescents with co-occurring ADHD and BPD would show the highest suicidality with a potential interaction effect (ADHD × BPD).

## 2. Materials and Methods

### 2.1. Participants

This cross-sectional observational clinical study included 108 Polish adolescents (66.7% female) aged 15.3 ± 1.3 years (range: 13–17 years), recruited from inpatient and outpatient units at the Independent Public Healthcare Facility, Children and Youth Treatment Center in Zabór, the Youth Sociotherapy Center (YSTC) No. 2 in Wrocław, and the District Educational Center in Jerzmanice-Zdrój (Poland). The data collection for our study was conducted between May 2024 and July 2025. No additional data were obtained outside this period.

Inclusion criteria were (a) sufficient understanding of the Polish language, (b) ability to comply with study procedures, and (c) met the diagnostic criteria for at least one psychiatric disorder, as determined using the Mini International Neuropsychiatric Interview for Children and Adolescents (MINI-KID) [21]. Exclusion criteria included (a) acute psychotic symptoms, (b) intellectual disability, and (c) any acute medical or psychiatric condition limiting the capacity to participate. Substance use disorders and exposure to child maltreatment were not used as exclusion criteria, as the study aimed to reflect a naturalistic clinical adolescent population in which such factors frequently co-occur with psychiatric disorders and suicidality. Participants represented a convenience sample, recruited from accessible inpatient and outpatient clinical settings.

The study protocol was approved by the Bioethics Committee of Wroclaw Medical University (Poland), approval no. KB452/2023N on 21 December 2023, and conducted in accordance with the ethical principles outlined in the 2013 Declaration of Helsinki. Written informed consent was obtained from both the participants and their legal guardians prior to participation.

### 2.2. Procedure

All assessments were conducted individually in a quiet clinical setting by trained psychiatrists. Each assessment session lasted approximately 60–90 min. During the session, the psychiatrist administered the MINI-KID 7.0.2 [21] to establish categorical psychiatric diagnoses (including ADHD and BPD) and to evaluate the participant’s suicidal risk using the suicidality module. Following the interview, participants received a brief debriefing, and if any acute suicidality was detected, immediate clinical follow-up was provided by the attending psychiatrist. All collected data were anonymized prior to statistical analysis.

### 2.3. The Mini International Neuropsychiatric Interview for Children and Adolescents (MINI-KID)

The MINI-KID is a structured diagnostic interview based on DSM-V and ICD-10 criteria, designed to assess psychiatric disorders in children and adolescents aged 6–17 years. It includes modules covering a broad range of mental disorders such as mood and anxiety disorders, ADHD, oppositional defiant and conduct disorders, eating disorders, tic disorders, obsessive–compulsive disorder, post-traumatic stress disorder (PTSD), substance use disorders, and suicidality. In this study, the Polish version of the MINI-KID 7.0.2 was used.

All MINI-KID interviews were administered by trained psychiatrists. In cases where diagnostic ambiguity occurred, final diagnoses were established through consensus between at least two qualified clinicians. Diagnostic data were coded as categorical variables (presence or absence of ADHD and BPD) for statistical analysis.

The MINI-KID applies full DSM-V diagnostic criteria and has demonstrated good inter-rater reliability and validity in child and adolescent samples. Although the categorical diagnosis of BPD in adolescence remains debated, converging evidence indicates that borderline personality disorder can be reliably and validly diagnosed in adolescents when full DSM criteria are applied and diagnoses are established through structured clinical interviews and careful clinical evaluation [20,22]. For the purposes of this study, borderline personality disorder was coded only when the full MINI-KID DSM-5 diagnostic algorithm was met (i.e., ≥5 endorsed criteria). Participants not meeting the full algorithm were coded as BPD-negative.

Suicidal risk was assessed using the suicidality module (Section B) of the MINI-KID. This module consists of structured yes-or-no items assessing the presence and severity of suicidal thoughts, planning, and attempts within the past month. Each affirmative response carries a severity weight, producing a total suicidality score ranging from 0 to 169. In the present study, the total suicidality score was treated as a continuous variable, reflecting the severity of suicidal risk.

The 19 MINI-KID items are rated with a yes-or-no scale and weighted according to their estimated contribution to suicidality. Eight items pertain to the past month and assess passive and active forms of suicidal ideation (3 items; Yes = 1, 4, and 6 points; 5 items; Yes = 8 points), four items pertain to suicide plans (Yes = 8 points), three items pertain to active preparations for a suicide attempt (Yes = 9, 10, and 11 points), and three items pertain to attempts in the past month (Yes = 12, 13, and 14 points). One item assesses whether the person has attempted suicide in their lifetime (Yes = 4 points), and one item assesses whether the person would like to attempt suicide in the future (Yes = 13 points). Internal consistency of the MINI-KID suicidality module in this clinical adolescent sample was excellent (Cronbach’s α = 0.91), reflecting consistent inter-item correlations within the suicidality module.

### 2.4. Statistical Analysis

All statistical analyses were conducted using the Statistica software, version 13.4.6.2 (TIBCO Software Inc., Palo Alto, CA, USA). Descriptive statistics (means, standard deviations, and frequencies) were calculated for all demographic and clinical variables.

To examine the associations between ADHD, BPD, and suicidality, a multistep regression strategy was employed, combining primary analyses with prespecified sensitivity and robustness analyses. Linear regression models were fitted with the MINI-KID suicidality total score as the dependent variable. Predictor variables included categorical diagnoses of ADHD (0 = absent, 1 = present), BPD (0 = absent, 1 = present), and their interaction term (ADHD × BPD). Analyses were conducted in two main stages: (1) unadjusted models and (2) adjusted models including a priori selected covariates (age, sex, current depressive episode, and use of psychiatric medications). These covariates were chosen based on consistent evidence identifying them as robust correlates of adolescent suicidality, independent of diagnostic status. To avoid overadjustment and loss of statistical power given the sample size, additional covariates were not included in the primary models. Because a substantial proportion of participants had a suicidality score of zero, the distribution of the MINI-KID suicidality score was positively skewed. Therefore, in addition to ordinary least squares (OLS) regression, two-part (hurdle-type) robustness analyses were conducted. First, logistic regression was used to model the presence of any suicidality (score > 0). Second, among participants with non-zero suicidality scores, predictors of suicidality severity were examined using OLS regression with heteroskedasticity-consistent (HC3) standard errors and robust regression with a Huber M-estimator to reduce the influence of outliers and departures from normality.

Logistic regression models were also used to examine predictors of the presence of any suicide risk as a complementary analytic approach. Predictor variables mirrored the linear models, and adjusted models included the same core covariates. To further assess the robustness of findings and address potential residual confounding by clinical severity and psychiatric comorbidity, a series of prespecified sensitivity analyses was conducted. These models sequentially adjusted for: (a) current depressive episode, (b) proxies of clinical severity including psychiatric medication use and treatment setting (inpatient vs. outpatient), and (c) additional comorbid diagnoses (current anxiety disorder and conduct disorder). The stability of ADHD and BPD coefficients across these models was examined to evaluate the consistency of results.

Prior to regression analyses, multicollinearity was assessed using the variance inflation factors (VIFs), and all VIF values below 2.0 indicated no significant multicollinearity. Missing data were minimal (<5%) and handled via listwise deletion. Statistical significance was set at *p* < 0.05. No a priori power analysis was conducted because recruitment followed clinical availability.

## 3. Results

### 3.1. Sample Characteristics

The study included 108 adolescents (66.7% female) aged 13–17 years (M = 15.3, SD = 1.3). All participants met criteria for at least one MINI-KID psychiatric diagnosis (Table 1). The most common disorders were depressive disorders, with a lifetime prevalence of 43.5% and a current episode prevalence of 26.9%, followed by current anxiety disorders (34%) and current behavioral conditions such as conduct disorder (18%) and oppositional defiant disorder (15%). ADHD was diagnosed in 29 participants (26.9%), BPD was diagnosed in 32 participants (29.6%), and 10 participants (9.3%) met criteria for both ADHD and BPD. Use of psychiatric medications was reported by 61.1% of participants, most commonly antidepressants (32.4%) and antipsychotics (26.9%). Suicidality (any score > 0 on the MINI-KID suicidality module) was present in 62.0% of adolescents, with a mean suicidality score of 38.3 ± 43.2 points.

### 3.2. Associations of ADHD and BPD with Suicide Risk

Linear regression models were conducted to examine associations between ADHD, BPD, and suicidality scores (Table 2). In the unadjusted model, both ADHD (β = 0.251, *p* = 0.024) and BPD (β = 0.397, *p* < 0.001) were significantly associated with higher suicidality. No significant ADHD × BPD interaction was found (β = −0.135, *p* = 0.286). After adjustment for age, sex, current depression, and use of psychiatric medications, the effect of BPD remained significant (β = 0.311, *p* = 0.004), whereas the effect of ADHD did not reach statistical significance (β = 0.204, *p* = 0.058). Among covariates, female sex (β = 0.187, *p* = 0.042) and use of psychiatric medications (β = 0.200, *p* = 0.025) were also significantly associated with higher suicidality scores, whereas age and current depressive episode were not (*p* > 0.1).

Residual diagnostics indicated deviations from normality of residuals, consistent with the zero-inflated distribution of suicidality scores. Therefore, two-part and robust regression analyses were conducted. Across these analyses, the direction and magnitude of effects were consistent with the primary linear and logistic regression results, with BPD remaining a robust predictor of suicidality severity and no evidence of an ADHD × BPD interaction (Table S1).

Logistic regression analyses (Table 3) further examined predictors of any suicide risk (score > 0). In unadjusted models, both ADHD (OR = 3.58, 95% CI [1.13–11.28], *p* = 0.029) and BPD (OR = 8.11, 95% CI [2.15–30.50], *p* = 0.002) predicted suicide risk. After adjustment for covariates, BPD remained a significant predictor (OR = 7.00, 95% CI [1.55–31.57], *p* = 0.011), while the effect of ADHD did not reach statistical significance (OR = 3.48, 95% CI [0.93–13.08], *p* = 0.065). The interaction between ADHD and BPD was non-significant. Among covariates, female sex (OR = 4.31, 95% CI [1.55–11.97], *p* = 0.005) and use of psychiatric medications (OR = 2.85, 95% CI [1.06–7.67], *p* = 0.038) were independently associated with higher suicide risk, whereas age and current depressive episode did not show significant associations (*p* > 0.05).

As shown in Table 4, the prevalence of any suicide risk was highest among adolescents with co-occurring ADHD and BPD (90%), followed by BPD-only (86.4%) and ADHD-only (73.7%) subgroups. Adolescents without either diagnosis had the lowest prevalence (43.9%). Mean suicidality scores followed a similar pattern, increasing from 22.6 in the ADHD−/BPD− group to 64.4 in the ADHD+/BPD+ group.

### 3.3. Sensitivity Analyses

Sensitivity analyses confirmed the robustness of the association between BPD and suicidality severity across all model specifications, including adjustment for depressive episode, psychiatric comorbidity, and proxies of clinical severity such as medication use and treatment setting. In contrast, the association between ADHD and suicidality severity was attenuated and no longer statistically significant after controlling for indicators of clinical severity, particularly residential treatment setting. No ADHD × BPD interaction emerged in any sensitivity model. Detailed results of these analyses, including models with additional covariates and robust regression approaches, are provided in the Supplementary Materials (Table S2).

## 4. Discussion

### 4.1. Main Findings

This study examined whether ADHD and BPD diagnoses show additive or interactive associations with suicidality in adolescents. Three main conclusions emerged. First, BPD was the strongest and independent diagnostic predictor of suicide risk even after adjustment for covariates. Second, ADHD showed an association with suicide risk only in unadjusted models; this association appeared not to be significant when age, sex, depression, and use of psychiatric medications were controlled for. Third, no evidence for a multiplicative increase was observed, although the study was underpowered to detect small interaction effects. Overall, the results suggest that adolescent suicidality is more strongly linked to BPD pathology than to ADHD, and that the association between ADHD and suicidality may depend on co-occurring clinical and demographic factors rather than reflecting ADHD-specific mechanisms. To our knowledge, this is the first study to directly test additive versus interactive effects of ADHD and BPD on acute suicide risk in adolescents using structured diagnostic interviews.

### 4.2. BPD as a Predictor of Suicide Risk

The strong association between BPD and suicidality in this sample aligns with extensive evidence identifying BPD as one of the most robust clinical predictors of suicidal thoughts and behaviors in youth [11,12]. Importantly, unlike many studies that rely on dimensional symptom measures, the current study used structured MINI-KID diagnostic interviews, providing precise and clinically validated diagnostic classification. The association between BPD and suicidality remained significant even after adjusting for current depression, suggesting that BPD is associated with unique, disorder-specific risk related to core features such as emotional instability, interpersonal hypersensitivity, and impulsive reactions to distress [20]. These findings reinforce BPD as a central clinical correlate for acute suicidality in clinical adolescent populations.

### 4.3. ADHD and Suicidality

In our unadjusted models, ADHD was significantly associated with greater suicidality, alongside BPD, consistent with meta-analytic literature showing elevated risk of suicidal thoughts and behaviors among youth with ADHD. A large meta-analysis of a heterogeneous population [8] confirmed the association between ADHD and suicidal behavior regardless of numerous mediators and moderators. In contrast, a recent meta-analysis [9] focused specifically on longitudinal adolescent samples and found an association but reported mixed and often inconclusive findings regarding moderators of the ADHD–suicidality link.

After adjustment for age, sex, current depression, and use of psychiatric medications, the association between ADHD and suicidality was no longer statistically significant. This attenuation suggests that the observed effect of ADHD in unadjusted analyses may be partly attributable to co-occurring depressive symptoms and treatment-related factors. Similarly, research [16,19] has highlighted that comorbid psychopathology—including borderline traits—can contribute to elevated suicidality in adolescents with ADHD. Taken together, these findings indicate that while ADHD is initially associated with suicide risk, its unique contribution is diminished when accounting for depression, treatment-related factors, and other accompanying clinical features.

However, the adjusted association between ADHD and suicidality should be interpreted as clinically suggestive but statistically uncertain rather than as evidence of no effect. Across linear, logistic, and robust analytic approaches, effect estimates consistently pointed in the same direction, yet confidence intervals were wide and conventional significance thresholds were not met after covariate adjustment, likely reflecting limited statistical power and residual confounding by clinical severity. Accordingly, these findings do not rule out a meaningful contribution of ADHD to suicidal risk in adolescents but suggest that its independent effect may be smaller, more context-dependent, and less robust than that observed for BPD.

### 4.4. Absence of an ADHD × BPD Interaction

No significant ADHD × BPD interaction emerged, indicating that the co-occurrence of both diagnoses did not multiplicatively increase acute suicide risk. Instead, the effects appeared largely additive. The pattern across ADHD × BPD subgroups shows that although adolescents with both diagnoses had the highest levels of suicide risk, the severity observed in the comorbid group reflected additive contributions of ADHD and BPD, not a synergistic interaction.

Although previous research [16] indicates that adolescents with comorbid ADHD × BPD exhibit more severe self-harm and suicidal ideation, such findings do not demonstrate a statistical interaction; rather, they underscore the central role of borderline pathology in shaping clinical severity.

### 4.5. Implications for Clinical Practice and Future Directions

The present findings highlight the central role of borderline pathology in assessing acute suicide risk in adolescents. Clinical evaluations should therefore include systematic screening for BPD features, even when adolescents present with primary diagnoses such as ADHD. Patterns observed across ADHD × BPD subgroups (Table 4) suggest that adolescents with comorbid presentations are at the highest risk, emphasizing the importance of evaluating both diagnoses in clinical practice.

Early identification of emotional instability, interpersonal sensitivity, and impulsive self-harm tendencies may support timely safety planning and targeted interventions focused on emotion regulation. Conversely, suicide risk among adolescents with ADHD appears to be substantially influenced by coexisting affective and clinical pathology, suggesting that clinicians should evaluate comorbid symptoms rather than assuming elevated risk based on ADHD alone.

Future research should employ longitudinal designs to clarify temporal pathways and potential mediators linking ADHD, BPD, and suicidality. Larger samples are needed to examine interaction effects with greater statistical power, particularly given the relatively low prevalence of full-threshold ADHD × BPD comorbidity. Studies incorporating trauma exposure, family dynamics, and treatment adherence will help build more comprehensive models of suicidal risk trajectories and may inform more precise risk stratification and prevention strategies.

### 4.6. Limitations

Certain limitations should be considered when interpreting the findings.

A first limitation is the absence of an a priori power analysis; therefore, the study may have been underpowered to detect interaction effects and the absence of a significant ADHD × BPD interaction should be interpreted cautiously. Consistent with this literature, clinical studies examining ADHD–BPD comorbidity in adolescents have highlighted the challenge of limited subgroup sizes when characterizing comorbid presentations, which constrains the feasibility of more complex statistical analyses, including formal tests of additive and interactive effects [15,16,17]. Accordingly, the small number of adolescents meeting criteria for both ADHD and BPD in the present study (*n* = 10) substantially limits power for interaction testing and underscores the need for larger, multi-site samples in future research.

Moreover, the diagnosis of borderline personality disorder in adolescents remains clinically and conceptually debated, as personality traits are still developing and core BPD features may overlap with normative adolescent emotional reactivity or with symptoms of other disorders such as depression or ADHD. Although structured interviews applying full DSM-5 criteria have demonstrated acceptable reliability and clinical utility in youth, the stability of categorical BPD diagnoses over time is limited. Consequently, findings related to BPD in adolescent samples should be interpreted as reflecting current patterns of severe emotion dysregulation rather than fixed personality pathology. The reliance on categorical diagnostic classifications represents an additional limitation, as this approach does not capture dimensional variation in ADHD and BPD symptom severity or subthreshold traits that are common in adolescence and strongly linked to suicidality. Future studies should incorporate dimensional measures of emotion dysregulation, impulsivity, and borderline traits to better characterize risk gradients and to examine whether interaction effects emerge at the symptom level rather than at the diagnostic level.

Furthermore, the cross-sectional design precludes conclusions regarding causal relationships among ADHD, BPD, and suicidality. Unmeasured contextual factors—such as trauma exposure, family functioning, treatment adherence, or environmental stress—may also have contributed to suicidal risk but were not assessed. In addition, substance use disorders and exposure to maltreatment were not excluded and were not modeled as covariates, which may have contributed to residual confounding; however, this approach enhances ecological validity by reflecting real-world clinical complexity.

Finally, the clinical convenience sampling approach limits generalizability to broader populations. Moreover, the regression models explained a modest proportion of variance (R^2^ = 0.16–0.23), indicating that additional, unmeasured factors likely contribute substantially to suicide risk.

## 5. Conclusions

BPD emerged as the strongest and independent predictor of suicidality in adolescents, underscoring the critical role of borderline pathology in clinical risk assessment. ADHD showed an association with suicidality only in unadjusted analyses; after covariate adjustment, this association was attenuated and did not reach conventional levels of statistical significance, suggesting that suicidal vulnerability in adolescents with ADHD may be partly attributable to co-occurring clinical and demographic factors. No statistically detectable ADHD × BPD interaction was observed; however, small interaction effects cannot be ruled out due to limited power. These findings emphasize the importance of early identification and targeted interventions addressing emotion regulation and interpersonal functioning to mitigate suicide risk in adolescents.

## Figures and Tables

**Table 1:** Demographic and clinical characteristics of the adolescent psychiatric sample (*N* = 108).

	*n* (%) or Mean ± SD
Age	15.30 ± 1.30
Sex, females	72 (66.7%)
ADHD (−), BPD (−)	57 (52.8%)
ADHD (+), BPD (−)	19 (17.6%)
ADHD (−), BPD (+)	22 (20.4%)
ADHD (+), BPD (+)	10 (9.3%)
Depression, lifetime history	47 (43.5%)
Depression, current episode	29 (26.9%)
Suicide risk, >0	67 (62.0%)
Suicide risk score ^1^	38.3 ± 43.2
Use of psychiatric medications	66 (61.1%)
Antidepressants	35 (32.4%)
Mood stabilizers	9 (8.4%)
Psychostimulants	15 (13.9%)
Antipsychotics	29 (26.9%)
Anxiolytics	8 (7.4%)
Other psychiatric medications ^2^	12 (11.1%)

Note: ADHD, attention-deficit/hyperactivity disorder; BPD, borderline personality disorder. ^1^ Suicide risk score is expressed in points (range 0–169) on the MINI-KID suicidality module. ^2^ Other psychiatric medications include pregabalin and atomoxetine.

**Table 2:** Linear regression models testing for the association of borderline personality disorder (BPD) and attention-deficit/hyperactivity disorder (ADHD) with suicide risk.

Model	Predictor	β	*p*
Unadjusted analysis (R^2^ = 0.163)	ADHD	0.251	**0.024**
	BPD	0.397	**<0.001**
	ADHD × BPD	−0.135	0.286
Adjusted analysis (R^2^ = 0.228)	ADHD	0.204	0.058
	BPD	0.311	**0.004**
	ADHD × BPD	−0.063	0.607
	Age	−0.001	0.988
	Sex, females	0.187	**0.042**
	Depression, current episode	0.135	0.137
	Use of psychiatric medications	0.200	**0.025**

Significant associations (*p* < 0.05) are bolded.

**Table 3:** Logistic regression analysis testing for the association of borderline personality disorder (BPD) and attention-deficit/hyperactivity disorder (ADHD) with suicide risk.

Model	Predictor	OR	95%CI	*p*
Unadjusted analysis (Nagelkerke R^2^ = 0.22)	ADHD	3.58	1.13–11.28	**0.029**
	BPD	8.11	2.15–30.50	**0.002**
	ADHD × BPD	0.40	0.02–5.65	0.495
Adjusted analysis (Nagelkerke R^2^ = 0.43)	ADHD	3.48	0.93–13.08	0.065
	BPD	7.00	1.55–31.57	**0.011**
	ADHD × BPD	0.57	0.03–9.99	0.703
	Age	0.94	0.62–1.48	0.836
	Sex, females	4.31	1.55–11.97	**0.005**
	Depression, current episode	3.32	0.98–11.26	0.054
	Use of psychiatric medications	2.85	1.06–7.67	**0.038**

Significant associations (*p* < 0.05) are bolded.

**Table 4:** Suicide risk across ADHD × BPD subgroups.

Group	*n*	Suicide Risk, >0 (%)	Suicide Risk Score (Mean ± SD)
ADHD (−), BPD (−)	57	43.9%	22.6 ± 36.1
ADHD (+), BPD (−)	19	73.7%	46.9 ± 41.0
ADHD (−), BPD (+)	22	86.4%	60.0 ± 49.8
ADHD (+), BPD (+)	10	90.0%	64.4 ± 37.0

## Data Availability

The anonymized dataset supporting the findings of this study is available from the corresponding author upon reasonable request. Although all data have been fully anonymized, they contain sensitive clinical information and therefore cannot be made publicly accessible.

## Supplementary Materials

The following supporting information can be downloaded at: https://www.mdpi.com/article/10.3390/jcm15010224/s1, Table S1: Robust linear regression (Huber M-estimator) predicting suicidality severity (MINI-KID Suicide risk score); Table S2: Sensitivity linear regression models (M1–M4) predicting suicidality severity (MINI-KID Suicide risk score) with robust (HC3) standard errors.

## Abbreviations

The following abbreviations are used in this manuscript:

ADHD	Attention-deficit/hyperactivity disorder
BPD	Borderline personality disorder
MINI-KID	Mini International Neuropsychiatric Interview for Children and Adolescents

## References

[1] Auerbach R.P., Stewart J.G., Johnson S.L. (2017). Impulsivity and suicidality in adolescent inpatients. J. Abnorm. Child Psychol..

[2] Hatkevich C., Penner F., Sharp C. (2019). Difficulties in emotion regulation and suicide ideation and attempt in adolescent inpatients. Psychiatry Res..

[3] Ayano G., Demelash S., Gizachew Y., Tsegay L., Alati R. (2023). The global prevalence of attention deficit hyperactivity disorder in children and adolescents: An umbrella review of meta-analyses. J. Affect. Disord..

[4] American Psychiatric Association (2013). Diagnostic and Statistical Manual of Mental Disorders.

[5] Ros R., Graziano P.A. (2018). Social functioning in children with or at risk for attention deficit/hyperactivity disorder: A metaanalytic review. J. Clin. Child Adolesc. Psychol..

[6] Njardvik U., Wergeland G.J., Riise E.N., Hannesdottir D.K., Öst L.G. (2025). Psychiatric comorbidity in children and adolescents with ADHD: A systematic review and metaanalysis. Clin. Psychol. Rev..

[7] Reale L., Bartoli B., Cartabia M., Zanetti M., Costantino M.A., Canevini M.P., Bonati M. (2017). Comorbidity prevalence and treatment outcome in children and adolescents with ADHD. Eur. Child Adolesc. Psychiatry.

[8] Septier M., Stordeur C., Zhang J., Delorme R., Cortese S. (2019). Association between suicidal spectrum behaviors and attention-deficit/hyperactivity disorder: A systematic review and meta-analysis. Neurosci. Biobehav. Rev..

[9] Garas P., Takacs Z.K., Balázs J. (2025). Longitudinal suicide risk in children and adolescents with attention deficit and hyperactivity disorder: A systematic review and meta-analysis. Brain Behav..

[10] Fonagy P., Speranza M., Luyten P., Kaess M., Hessels C., Bohus M. (2015). ESCAP Expert Article: Borderline personality disorder in adolescence: An expert research review with implications for clinical practice. Eur. Child Adolesc. Psychiatry.

[11] Moselli M., Casini M.P., Frattini C., Williams R. (2023). Suicidality and personality pathology in adolescence: A systematic review. Child Psychiatry Hum. Dev..

[12] Sękowski M., Gambin M., Sumlin E., Sharp C. (2022). Associations between symptoms of borderline personality disorder and suicidality in inpatient adolescents: The significance of identity disturbance. Psychiatry Res..

[13] Bernardi S., Faraone S.V., Cortese S., Kerridge B.T., Pallanti S., Wang S., Blanco C. (2012). The lifetime impact of attention-deficit hyperactivity disorder: Results from the National Epidemiologic Survey on Alcohol and Related Conditions. Psychol. Med..

[14] Kuja-Halkola R., Lind Juto K., Skoglund C., Rück C., Mataix-Cols D., Pérez-Vigil A., Larsson J., Hellner C., Långström N., Petrovic P. (2021). Do borderline personality disorder and attention-deficit/hyperactivity disorder co-aggregate in families? A population-based study of 2 million Swedes. Mol. Psychiatry.

[15] Korsgaard H.O., Torgersen S., Wentzel-Larsen T., Ulberg R. (2016). Personality disorders and Axis I comorbidity in adolescent outpatients with ADHD. BMC Psychiatry.

[16] Akça Ö.F., Wall K., Sharp C. (2020). Borderline personality disorder and attention deficit/hyperactivity disorder in adolescence: Overlap and differences in a clinical setting. Borderline Pers. Emot. Dysregul..

[17] Speranza M., Revah-Levy A., Cortese S., Falissard B., Pham-Scottez A., Corcos M. (2011). ADHD in adolescents with borderline personality disorder. BMC Psychiatry.

[18] Prada P., Hasler R., Baud P., Bednarz G., Ardu S., Krejci I., Nicastro R., Aubry J.-M., Perroud N. (2014). Distinguishing borderline personality disorder from adult attention deficit/hyperactivity disorder: A clinical and dimensional perspective. Psychiatry Res..

[19] Blay M., De Godoy F., Hasler R., Nicastro R., Pham E., Weibel S., Perroud N. (2025). Is adult attention deficit hyperactivity disorder associated with non-suicidal self-injury and/or suicidal behaviors? A study on the role of borderline personality disorder comorbidity. J. Affect. Disord..

[20] Sharp C., Fonagy P. (2015). Practitioner Review: Borderline personality disorder in adolescence-recent conceptualization, intervention, and implications for clinical practice. J. Child Psychol. Psychiatry.

[21] Sheehan D.V., Sheehan K.H., Shytle R.D., Janavs J., Bannon Y., Rogers J.E., Milo K.M., Stock S.L., Wilkinson B. (2010). Reliability and validity of the mini international neuropsychiatric interview for children and adolescents (MINI-KID). J. Clin. Psychiatry.

[22] Chanen A.M., Nicol K., Betts J.K., Thompson K.N. (2020). Diagnosis and Treatment of Borderline Personality Disorder in Young People. Curr. Psychiatry Rep..

