# Supplementary material for: Associations of ADHD and Borderline Personality Disorder with Suicidality in Adolescents: Additive and Interactive Effects
Source: J Clin Med. 2025 Dec 27;15(1):224. doi: 10.3390/jcm15010224 (PMC12787169; doi:10.3390/jcm15010224)
Supplement: Supplementary file 1 [file jcm-15-00224-s001.zip › jcm-4043461-supplementary.pdf]

1. Supplementary Materials

The Supplementary Materials provide detailed results of prespecified sensitivity and robustness analyses conducted to examine the stability of the associations between attention-deficit/hyperactivity disorder (ADHD), borderline personality disorder (BPD), and suicidality severity in adolescents. These analyses were performed to assess the influence of additional covariates reflecting psychiatric comorbidity and clinical severity, as well as to evaluate the robustness of findings to violations of normality and heteroskedasticity assumptions.

1.1 Robust linear regression

Table S1 presents results from a robust linear regression model using a Huber M-estimator to reduce the influence of outliers and departures from normality in suicidality scores. The model included ADHD, BPD, their interaction term (ADHD × BPD), and the same covariates as in the adjusted primary analysis. Robust heteroskedasticity-consistent (HC3) standard errors are reported.

Supplementary Table S1. Robust linear regression (Huber M-estimator) predicting suicidality severity (MINI-KID Suicide risk score)

**Predictor**	**β**	**Robust SE**	**p**
ADHD	22.07	11.42	0.053
BPD	30.94	13.85	**0.026**
Age	0.38	3.05	0.900
Sex, females	19.93	8.32	**0.017**
Depression, current episode	6.64	12.54	0.596
Psychiatric medications	17.81	8.74	**0.042**
ADHD × BPD	−6.21	23.76	0.794

Outcome variable: MINI-KID suicidality total score (range 0–169).
Sample size: N = 108.
Statistical notes: Regression coefficients are reported as unstandardized estimates with robust (HC3) standard errors.

1.2. Sensitivity linear regression models

Table S2 presents a series of prespecified sensitivity linear regression models examining the robustness of the associations between ADHD, BPD, and suicidality severity. All models were adjusted for age and sex. Model M1 included age and sex only. Model M2 additionally adjusted for current depressive episode. Model M3 further included proxies of clinical severity, namely psychiatric medication use and treatment setting (inpatient vs. outpatient). Model M4 additionally adjusted for current anxiety disorder and conduct disorder. Robust heteroskedasticity-consistent (HC3) standard errors were used in all models. Regression coefficients are reported as unstandardized β estimates.

Table S2. Sensitivity linear regression models (M1–M4) predicting suicidality severity (MINI-KID Suicide risk score) with robust (HC3) standard errors

Model	Predictor	β	Robust SE	p
M1 (age, sex)	ADHD	20.16	10.23	0.052
	BPD	30.76	11.56	**0.009**
	ADHD × BPD	−12.04	20.63	0.561
M2 (+ depression)	ADHD	21.76	10.45	**0.040**
	BPD	28.83	11.40	**0.013**
	ADHD × BPD	−9.52	19.88	0.633
M3 (+ psychiatric medications, treatment setting)	ADHD	6.22	9.28	0.504
	BPD	22.78	9.32	**0.016**
	ADHD × BPD	−1.05	15.09	0.945
M4 (+ anxiety, conduct disorder)	ADHD	21.94	10.86	**0.046**
	BPD	28.38	11.52	**0.015**
	ADHD × BPD	−8.83	21.10	0.677

Outcome variable: MINI-KID suicidality total score (range 0-169).

Statistical notes: Robust standard errors (HC3) are reported. Statistically significant effects ($p < 0.05$) are indicated in bold.
